# Situs inversus totalis and lung cancer: a case report of surgical resection after neoadjuvant chemoimmunotherapy for stage IIIB squamous cell carcinoma

**DOI:** 10.3389/fonc.2026.1756152

**Published:** 2026-04-20

**Authors:** Wenhao Wang, Zhouyi Lu, Wan Posum, Yifeng Qian, Haoxin Liu, Kaile Jiang, Xiaofeng Chen

**Affiliations:** 1 Department of Thoracic Surgery, Huashan Hospital & Cancer Metastasis Institute, Fudan University, Shanghai, China; 2 Department of Radiology, Huashan Hospital, Fudan University, Shanghai, China

**Keywords:** advanced stage, chemoimmunotherapy, lung squamous cell carcinoma, pembrolizumab, situs inversus totalis

## Abstract

Immune checkpoint inhibitors (ICIs) have emerged as a promising therapeutic strategy for lung cancer. This case report describes a patient with situs inversus totalis (SIT) and initially unresectable, advanced lung squamous cell carcinoma (LSCC) who successfully underwent surgical resection following neoadjuvant therapy with pembrolizumab and chemotherapy. The patient was incidentally found to have SIT and a 6.5 × 5.0 cm mass in the right upper lobe (RUL) with hilar and mediastinal lymphadenopathy on a local chest CT scan. PET-CT scan revealed the RUL lesion (approximately 6.6 cm, SUVmax 13.7) and multiple hypermetabolic lymph nodes in the right hilum and station 4R (largest 1.8×1.8 cm, SUVmax 2.5-5.2). EBUS/TBNA was not performed due to limited availability at the initial treating institution. The tumor was deemed unresectable due to its large size (6.6 cm), central location involving the apical segment bronchus, and bulky N2 disease (station 4R, >2 cm), which increased the risk of incomplete resection and recurrence. A CT-guided biopsy confirmed squamous cell carcinoma. The patient was staged as cT3N2M0 (stage IIIB) and received three cycles of neoadjuvant pembrolizumab (200 mg), cisplatin (60 mg), and paclitaxel (270 mg). A repeat PET-CT at our institution showed significant reduction of the tumor (approximately 1.9×1.7 cm, SUVmax 8.9) and mediastinal (stations 3A, 4) and hilar lymph nodes with decreased metabolic activity (SUVmax 2.5). The restaging was ycT1cN1M0 (stage IIA). Following a multidisciplinary team (MDT) discussion, the patient underwent video-assisted thoracoscopic surgery (VATS) right upper lobectomy with mediastinal lymph node dissection. The final pathology confirmed a pathological complete response in the lymph nodes and minimal residual disease in the primary lesion, yielding a postoperative pathological stage of ypT1cN0M0 (stage IA3). Postoperative management included supportive care, infection control, and airway clearance. The patient was discharged on postoperative day 4 and subsequently received adjuvant pembrolizumab monotherapy. This case demonstrates that neoadjuvant chemoimmunotherapy can effectively downstage initially unresectable advanced LSCC, even in a patient with SIT, creating an opportunity for successful surgical intervention and potentially improving outcomes, thereby informing future clinical practice.

## Background

Situs inversus totalis (SIT) is a rare autosomal recessive condition characterized by a 180° transposition of thoracic and abdominal viscera, presenting a mirror-image of the normal anatomy (1). The clinical reporting of lung cancer concurrent with SIT is exceedingly rare. In such cases, the anomalous anatomy poses unique challenges for radiographic interpretation, surgical planning, and operative technique. Lung squamous cell carcinoma (LSCC) ranks among the most common malignant pulmonary tumors. While early-stage LSCC can be effectively managed with surgery, advanced disease is often unresectable and carries a poor prognosis. Several phase III clinical trials (2–6) have demonstrated that combining immune checkpoint inhibitors (ICIs) with chemotherapy improves overall survival (OS) compared to chemotherapy alone. Neoadjuvant therapy can induce tumor shrinkage in some patients with locally advanced LSCC, potentially rendering them eligible for surgical resection. This report presents, to our knowledge, the first case of a patient with SIT and initially unresectable stage IIIB LSCC managed with neoadjuvant chemoimmunotherapy and subsequent surgery. A chest CT scan revealed a 6.5 × 5.0 cm tumor with hilar and mediastinal lymphadenopathy. Following three cycles of pembrolizumab combined with chemotherapy, the tumor significantly regressed, achieving a partial response (PR) and successful downstaging, which allowed for successful surgical intervention. The patient subsequently received adjuvant pembrolizumab maintenance therapy. This case highlights the potential of chemoimmunotherapy to convert unresectable LSCC, even in the setting of SIT, into an operable condition.

## Case presentation

**Figure 1:**
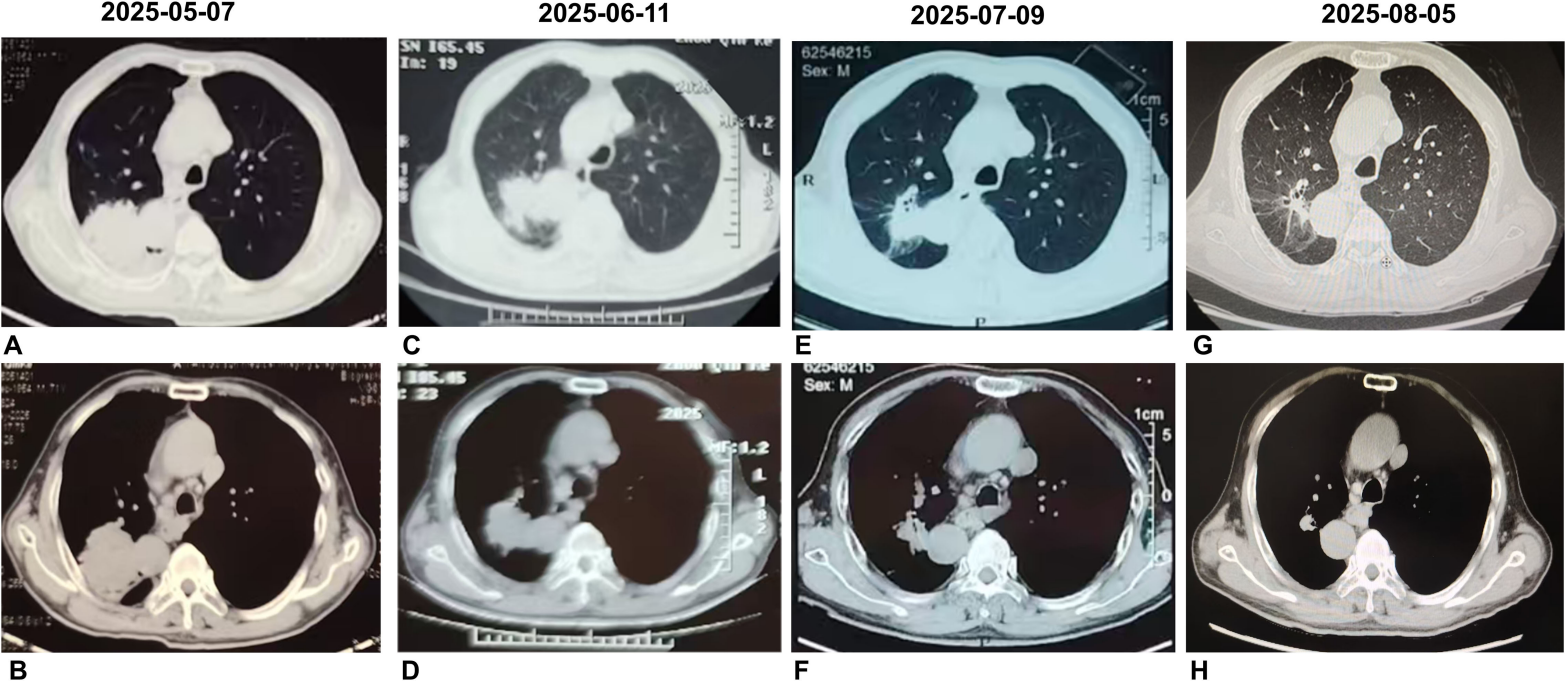
Imaging response to neoadjuvant chemoimmunotherapy. Initial chest CT **(A, B)** shows a 6.5 × 5.0 cm mass in the right upper lobe with hilar and mediastinal lymphadenopathy. After one **(C, D)**, two **(E, F)**, and three cycles **(G, H)** of treatment, the tumor progressively decreased in size. Following three cycles, the lesion measured 2.1 cm × 1.7 cm, with marked regression of the mediastinal and hilar nodes.

**Figure 2:**
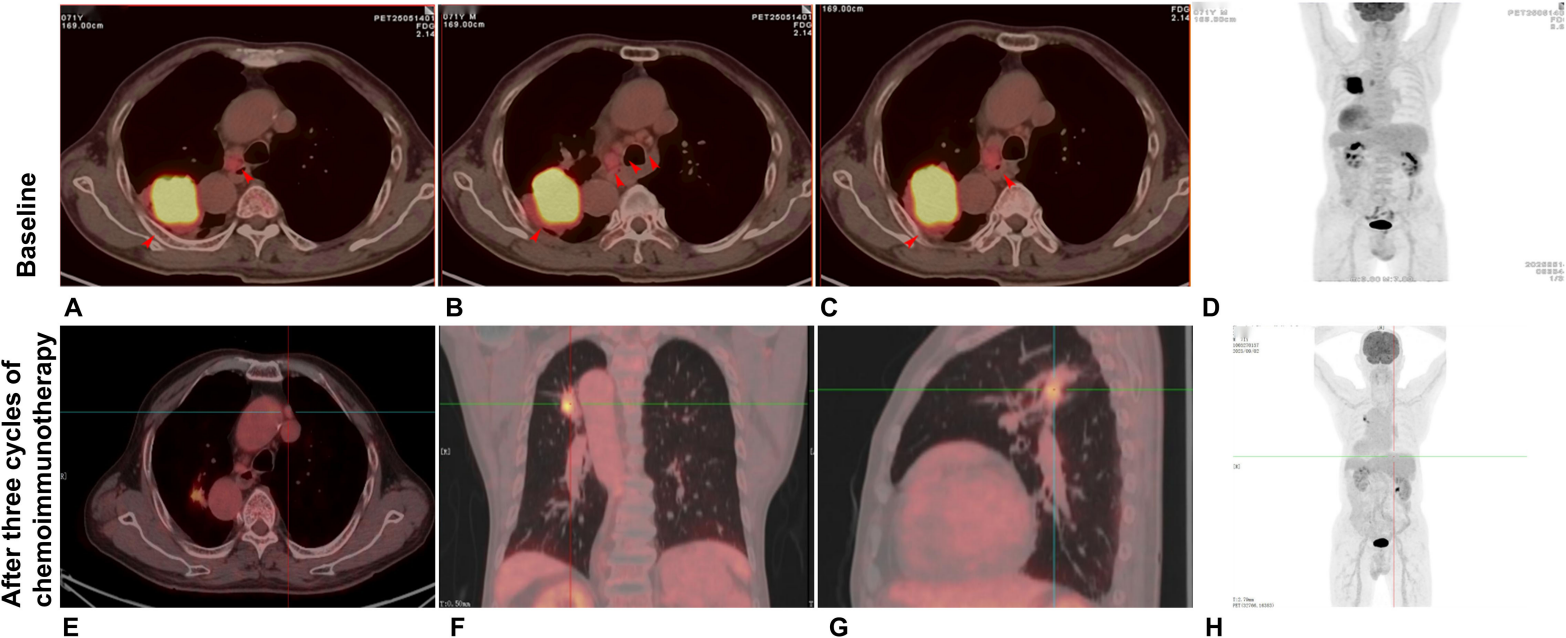
PET-CT comparison before and after neoadjuvant therapy. Initial PET-CT **(A–D)** reveals a 6.6 cm × 5.2 cm right upper lobe mass (SUVmax 13.7) with hypermetabolic hilar and mediastinal lymph nodes. Post-treatment imaging **(E–H)** demonstrates significant reduction of the primary lesion to 2.1 cm × 1.7 cm (SUVmax 8.9) and decreased size and metabolic activity of the nodal stations.

A 71-year-old male was admitted to our hospital with a confirmed diagnosis of lung cancer for 4 months. He had a smoking history of over 30 years. His weight was 65 kg, height 169 cm, yielding a body mass index (BMI) of 22.28 kg/m². There was no family history of malignancy or hereditary diseases, and no other significant physical examination findings were noted. In May 2025, a chest CT scan performed at a local hospital revealed SIT and a mass measuring 6.5 × 5.0 cm in the apicoposterior segment of the right upper lobe, accompanied by hilar and mediastinal lymphadenopathy, highly suggestive of malignancy. Subsequent bronchoscopy demonstrated stenosis in the apicoposterior segment of the right upper lobe (RUL). A CT-guided percutaneous biopsy was performed, which confirmed the diagnosis of LSCC. PD-L1 testing performed on the initial biopsy specimen revealed a tumor proportion score (TPS) of 5%. Baseline imaging and first-line treatment evaluation are summarized in **Figure 1**. A PET-CT scan conducted on May 14, 2025, showed a lobulated soft tissue mass (approximately 6.6×5.2 cm) in the apicoposterior segment of the RUL, causing obstructive changes in the corresponding bronchus and adhering to the adjacent pleura. The lesion exhibited markedly increased radiotracer uptake with a maximum standardized uptake value (SUVmax) of 13.7. Multiple enlarged lymph nodes with increased metabolic activity were identified in the right hilum and mediastinal station 4, the largest measuring about 1.8×1.8 cm with SUVmax values ranging from 2.5 to 5.2 (**Figures 2A–D**). EBUS/TBNA was not performed due to limited availability at the initial treating institution. The tumor was deemed unresectable due to its large size (6.6 cm), central location involving the apical segment bronchus, and bulky N2 disease which increased the risk of incomplete resection and recurrence. Based on the imaging and clinical findings, the patient was staged as cT3N2M0 (stage IIIB). After thorough discussion of the treatment plan and associated risks, the patient was initiated on a combination therapy regimen consisting of chemotherapy (Cisplatin: 60 mg; Paclitaxel: 270 mg) and immunotherapy (Pembrolizumab: 200 mg). The patient tolerated the three cycle neoadjuvant chemoimmunotherapy well without significant adverse effects.

Following the completion of three cycles of neoadjuvant chemoimmunotherapy, the patient’s tumor was evaluated as having achieved a partial response (PR). With the patient expressing a strong desire for surgical intervention, and considering the primary local hospital had declined due to the high surgical complexity associated with the anatomical anomalies, he was subsequently referred to our institution one month later for surgery. Upon admission, laboratory tests at our hospital revealed no abnormalities. A follow-up PET-CT scan (**Figures 2E–H**) demonstrated a significant reduction in the primary lesion, now presenting as an irregular hypermetabolic nodule in the apicoposterior segment of the RUL, measuring approximately 2.1 cm × 1.7 cm with a SUVmax of 8.9. This represented a marked decrease from the initial baseline measurement of 6.6 × 5.2 cm and SUVmax of 13.7. Furthermore, the previously noted mediastinal (stations 3A, 4) and right hilar lymph nodes had decreased in size and showed reduced metabolic activity, with a current SUVmax of 2.5. Based on these findings, the preoperative restaging was determined to be ycT1cN1M0 (stage IIA).

Besides, a comprehensive preoperative assessment was meticulously performed. Key preoperative evaluations included a transthoracic echocardiogram, which confirmed mirror-image dextrocardia, revealed moderate-to-severe aortic regurgitation, left ventricular wall thickening, significant left atrial and ventricular enlargement, and dilatation of the aortic root and ascending aorta, with a preserved left ventricular ejection fraction of 56%. Pulmonary function tests showed a forced vital capacity (FVC) of 2.64 L (70.7% of predicted) and a forced expiratory volume in one second (FEV1) of 1.73 L (59.4% of predicted). Arterial blood gas analysis indicated a PaCO_2_ of 36.5 mmHg, PaO_2_ of 71.2 mmHg, and an oxygen saturation of 93.5%. Notably, the patient’s functional capacity was assessed as being able to climb four flights of stairs. After a formal multidisciplinary team (MDT) consultation involving specialists from thoracic surgery, pulmonology, cardiac surgery, cardiology, and anesthesiology, a collective decision was made to proceed with surgery. Consequently, the patient successfully underwent a video-assisted thoracoscopic surgery (VATS) right upper lobectomy in August 2025.

**Figure 3:**
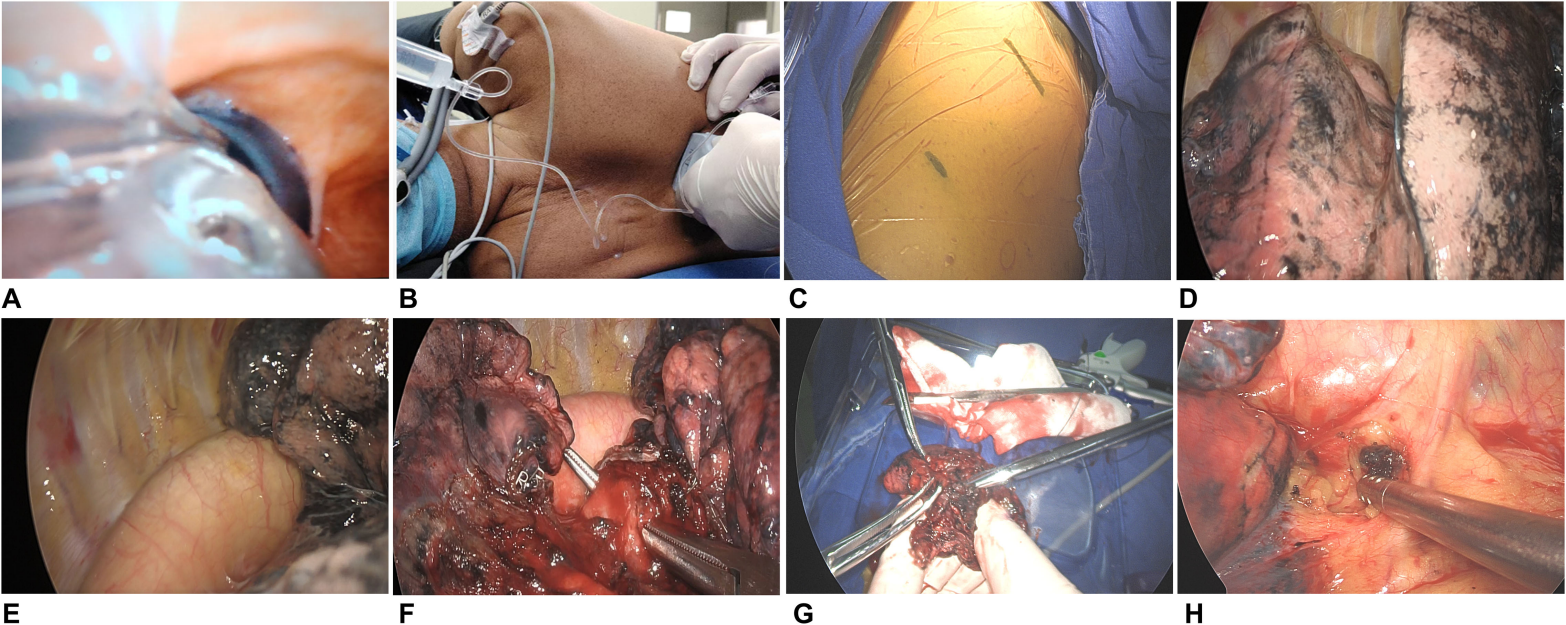
Intraoperative findings in situs inversus totalis. **(A)** Right-sided double-lumen intubation. **(B)** Preoperative subdural block. **(C)** The utility port was placed in the 4th intercostal space along the anterior axillary line, and the observation port in the 7th intercostal space along the midaxillary line. **(D)** Intraoperative exploration identified a right lung consisting of two lobes, with the tumor located in the right upper lobe. **(E)** Dextroposition of the heart and aorta. **(F)** Division of the right upper lobe vessels and bronchus. **(G)** The tumor measured approximately 2.2 cm in size. **(H)** Dissection of station 5 lymph nodes.

The patient underwent general anesthesia with a right-sided double-lumen endotracheal tube (**Figure 3A**) and preoperative subdural nerve block (**Figure 3B**). The patient was positioned in the lateral decubitus position, and the operative field was prepared and draped routinely. The utility port was established in the 4th intercostal space at the anterior axillary line, and the observation port was placed in the 7th intercostal space at the mid-axillary line (**Figure 3C**). Intraoperative exploration confirmed the presence of only two lobes in the right lung (upper and lower). A firm, approximately 2 cm nodule was identified in the right upper lobe. Mirror-image anatomy was observed: the right upper lobe bronchus, artery, and vein exhibited the branching pattern typically found in a normal left upper lobe (**Figure 3D**). The heart and aorta were located on the right side (**Figure 3E**). The major fissure was hypoplastic. The procedure involved dividing the incomplete oblique fissure with a linear stapler (Weray, China). The right upper lobe arteries, veins, and bronchus were sequentially dissected and transected with staplers (**Figure 3F**). The specimen was retrieved using a disposable specimen retrieval bag. The resected tumor measured approximately 2.2 cm, and the bronchial stump margin was sent for intraoperative pathological evaluation (**Figure 3G**). A systematic mediastinal lymph node dissection was performed, encompassing stations 5, 6, 7, 10, 11, and 12 (**Figure 3H**). After saline irrigation and an underwater leak test that showed no air leak, hemostasis was achieved. A single 24-F chest tube was inserted, and the incisions were closed. The total intraoperative blood loss was 100 mL, and the operative time was 55 minutes. The patient was transferred to the ward in stable condition. Postoperatively, intravenous antibiotics and mucolytic therapy were administered. The patient’s recovery was uneventful. The daily chest tube drainage volumes were 200 mL, 150 mL, and 50 mL on postoperative days 1–3, respectively. A chest X-ray (**Supplementary Figure 2A**) demonstrated satisfactory lung expansion. The chest tube was removed on postoperative day 4, and the patient was discharged.

The final pathologic examination (**Supplementary Figure 1**) established a postoperative stage of ypT1cN0M0 (Stage IA3). Histopathology confirmed a moderately differentiated, non-keratinizing squamous cell carcinoma measuring 2.2 cm × 2.2 cm × 1.8 cm. There was no evidence of pleural involvement, lymphovascular invasion, perineural invasion, or involvement of the surgical margins. All examined lymph nodes from stations 5, 6, 7, 10, 11, and 12 were negative for metastasis. Immunohistochemical staining was positive for P63, P40, P53, and CK. The tumor was negative for TTF-1, Syn, and NapsinA. The Ki-67 proliferation index was approximately 60%, and C-MET expression was observed in 70% of tumor cells. PD-L1 testing (clone E1L3N) revealed a tumor proportion score (TPS) of 5%. The resected primary tumor showed minimal residual viable tumor cells with approximately 5%. Subsequent genetic testing was negative for driver mutations.

On September 20, 2025, the patient commenced adjuvant immunotherapy with pembrolizumab 200 mg intravenously on day 1 every 3 weeks. At the time of writing, three cycles had been completed with excellent tolerance; no immune-related adverse events ≥ grade 2 have occurred. Serial surveillance of tumor markers, cardiac enzymes and thyroid function has remained within normal limits. The most recent radiographic evaluation showed no evidence of disease recurrence (**Supplementary Figures 2B–D**). At 3-month follow-up, the patient reported good functional recovery, with the ability to climb four flights of stairs without dyspnea. Formal pulmonary function testing was not repeated. The plan is to continue the adjuvant pembrolizumab per the standard protocol.

## Discussion

Approximately 30% of patients with non-small cell lung cancer (NSCLC) are diagnosed at a locally advanced stage (IIIA or IIIB). While radical surgery remains the preferred treatment for these patients, both resectability and the risk of postoperative relapse are major clinical concerns (7). Historical data indicate that platinum-based chemotherapy as a neoadjuvant approach provides only a modest 5% improvement in long-term survival, highlighting a substantial unmet clinical need (8).

The advent of PD-1/PD-L1 inhibitors has recently redefined the therapeutic landscape (9). For patients with unresectable, locally advanced NSCLC, consolidation durvalumab after concurrent chemoradiotherapy improves 3-year OS from 44% to 57% (10). These findings have established immunotherapy as the standard guideline for patients with locally advanced LSCC, particularly those without driver mutations.

However, while advancements for locally advanced LSCC are evident, the efficacy of these treatments in patients with concurrent SIT remains unreported. SIT is a rare condition characterized by the transposition of thoracic and abdominal viscera. Genetically, it is often an autosomal recessive trait but may also be associated with X-linked defects (11). The primary challenge in operating on patients with SIT stems from anatomical variations, which complicate both anesthetic management and surgical technique. To our knowledge, this report represents the first case worldwide of a patient with SIT and locally advanced LSCC successfully undergoing surgical resection after neoadjuvant chemoimmunotherapy. This case integrates complexities across anatomy, imaging, pulmonology, oncology, cardiology, anesthesiology, and thoracic surgical technique. The ACCP guidelines recommend a MDT approach for patients requiring complex, multimodal care (12). Our patient’s management adhered to this principle: pulmonologists performed the CT-guided biopsy for pathological confirmation, oncologists determined the neoadjuvant chemoimmunotherapy regimen, radiologists provided precise preoperative CT evaluations, and thoracic surgeons dynamically assessed surgical timing based on treatment response and successfully performed a VATS right upper lobectomy. The presence of SIT significantly increased procedural complexity. Our experience offers several key insights: Surgeons must possess an in-depth understanding of the altered anatomy, meticulously reviewing the mirror-image relationships and potential variations of the pulmonary arteries, veins, and bronchi preoperatively. Intraoperatively, the right upper lobe vasculature mirrored that of a typical left upper lobe, requiring the surgeon to maintain constant spatial reorientation. The apicoposterior artery arose as the first branch, similar to a left upper lobe. Mediastinal lymph node dissection was performed with heightened awareness of the right-sided aorta and the mirrored course of the recurrent laryngeal nerve. Anesthesia management was also complex due to the mirror-image bronchial anatomy; right-sided double-lumen intubation was successfully achieved under fiberoptic bronchoscopy guidance.

In this case, three cycles of neoadjuvant chemoimmunotherapy reduced the tumor’s maximum diameter from 6.6 cm to 1.9 cm. Notably, this downstaging was achieved without inducing significant pleural adhesions or hilar tissue edema and fibrosis, which are common challenges post-neoadjuvant therapy and can complicate surgery. This successful tumor reduction created a favorable window for subsequent radical resection. Although the procedure demanded advanced anatomical expertise and skills, we successfully completed the VATS lobectomy, demonstrating the feasibility and safety of this minimally invasive approach in patients with SIT.

The KEYNOTE-671 trial (13), a global phase III study, evaluated perioperative pembrolizumab (neoadjuvant pembrolizumab plus platinum-based chemotherapy followed by adjuvant pembrolizumab) in resectable stage II-IIIB NSCLC. The results showed a significant improvement in OS, with an estimated 36-month OS rate of 71% in the pembrolizumab group, alongside significantly higher rates of major pathological response (MPR) and pathological complete response (pCR). Our patient’s treatment pathway aligns with this evolving standard; after evaluation confirmed initial unresectability complicated by SIT, he received three cycles of neoadjuvant paclitaxel, carboplatin, and pembrolizumab. This successful conversion to resectability and the subsequent finding of no residual nodal disease on final pathology suggest that the chemoimmunotherapy regimen effectively induced nodal downstaging, consistent with research highlighting the pronounced effect of immunotherapy on metastatic lymph nodes (14, 15). Furthermore, the patient has maintained a stable condition with no recurrence or distant metastasis on repeated evaluations and has experienced no immune-related adverse events from the initiation of pembrolizumab through adjuvant maintenance, corroborating the feasibility and safety profile of this combination, even in patients with SIT.

## Conclusion

In summary, we present a case of locally advanced LSCC with SIT that was successfully managed with a multimodal approach. This case highlights two critical points: first, the MDT collaborative framework is fundamental for the precise management of complex cases, ensuring accurate staging and safe treatment despite anatomical challenges. Second, neoadjuvant chemoimmunotherapy demonstrates significant potential for converting initially unresectable tumors into operable ones. The co-occurrence of SIT markedly heightened the complexity and rarity of this presentation. We advocate systematic adoption of multidisciplinary planning and perioperative immunotherapy for all fit LSCC patients with locally advanced stage, irrespective of SIT anomalies.

## Data Availability

The original contributions presented in the study are included in the article/**Supplementary Material**. Further inquiries can be directed to the corresponding author.
