# Supplementary material for: Situs inversus totalis and lung cancer: a case report of surgical resection after neoadjuvant chemoimmunotherapy for stage IIIB squamous cell carcinoma
Source: Front Oncol. 2026 Apr 20;16:1756152. doi: 10.3389/fonc.2026.1756152 (PMC13135998; doi:10.3389/fonc.2026.1756152)
Supplement: Supplementary file 1 [file Table1.docx]

**[Supplementary M](https://www.frontiersin.org/journals/immunology/articles/10.3389/fimmu.2023.1328005/full#hsm)aterials**

**
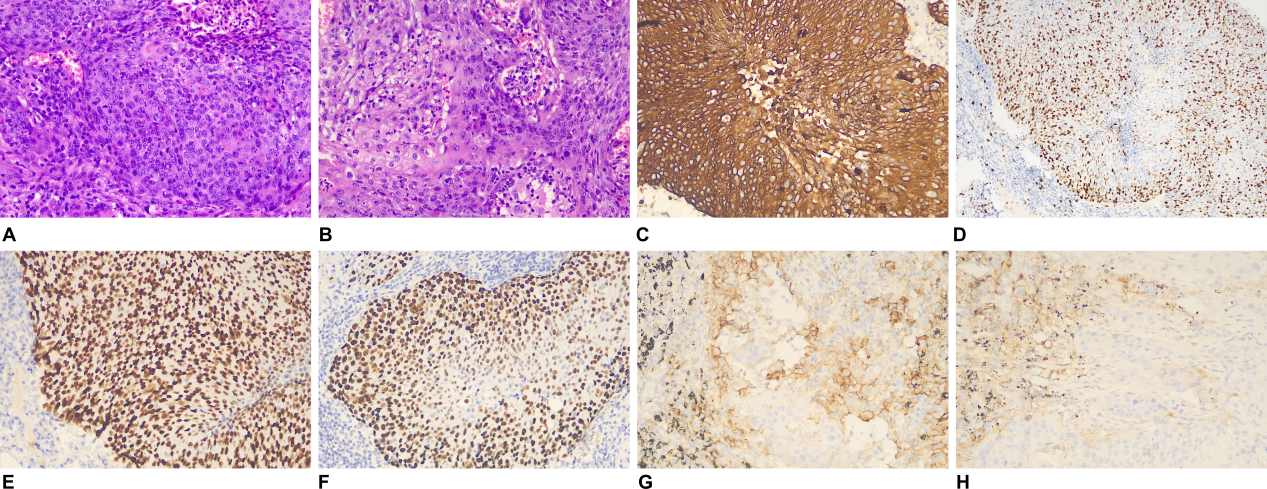
**

**[Supplementary Figure 1](https://www.frontiersin.org/journals/immunology/articles/10.3389/fimmu.2023.1328005/full#hsm).** Postoperative pathological confirmation of squamous cell carcinoma in the right upper lobe.

(A) Hematoxylin and eosin (H&E) staining, 100×. (B) H&E staining, 400×. (C–H) Immunohistochemical staining, 400×, demonstrating positive expression for CK (C), Ki67 (60% positive) (D), P40 (E), and P53 (F). Staining for PD1 (G) and PDL1 (H) is also shown.


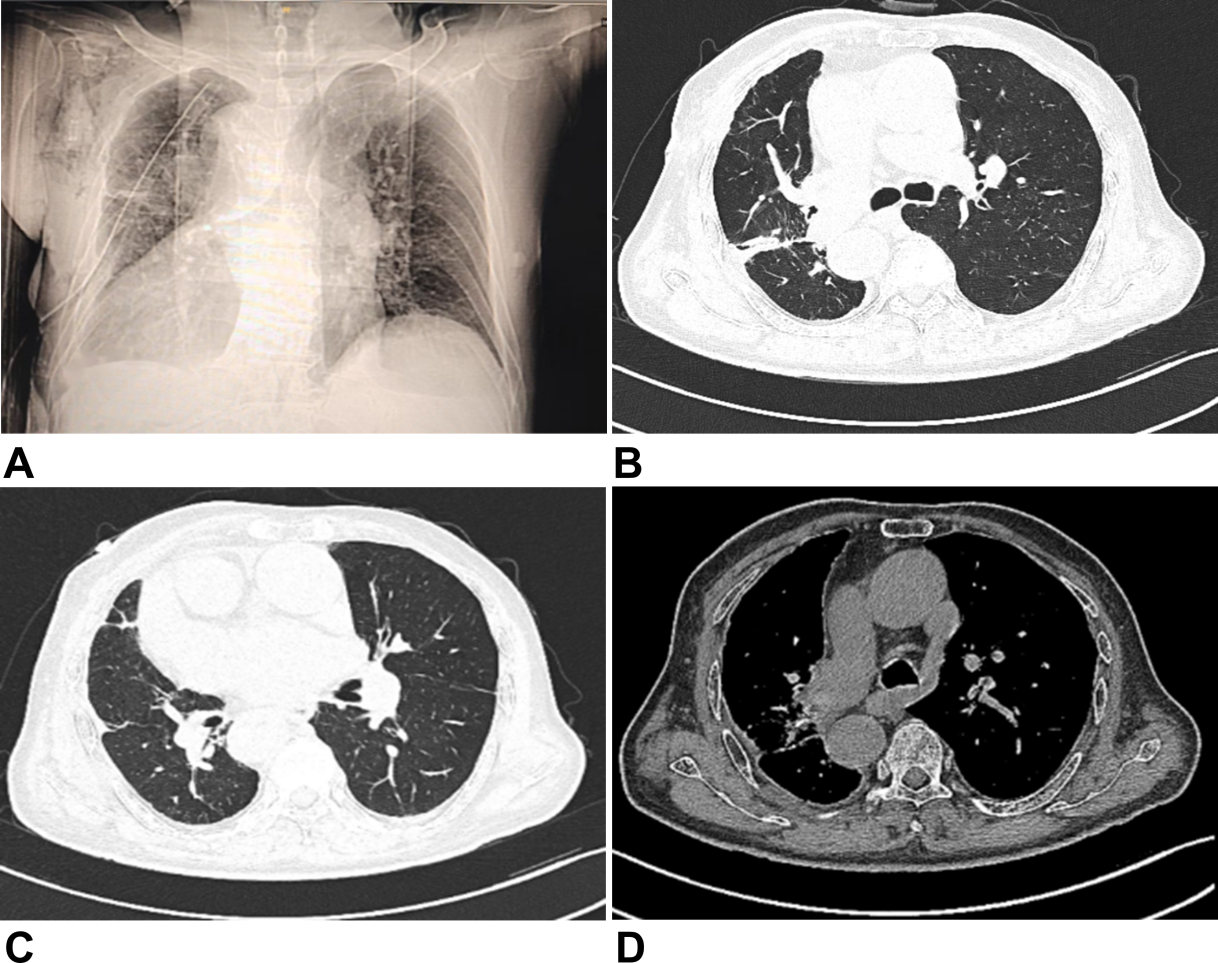


**[Supplementary Figure 2](https://www.frontiersin.org/journals/immunology/articles/10.3389/fimmu.2023.1328005/full#hsm).** Post-operative imaging.

The postoperative chest radiograph (A) shows satisfactory lung expansion. The most recent imaging examination (2025.11.6) revealed no evidence of tumor recurrence (B-D).
